# Bilateral Renal Infarcts Due to Blunt Trauma in a Healthy Young Female Patient: A Case Report

**DOI:** 10.7759/cureus.83366

**Published:** 2025-05-02

**Authors:** Taylor Locklear, Alyssa McMandon, Rachel A Daley, Saptarshi Biswas

**Affiliations:** 1 Surgery, Grand Strand Medical Center, Myrtle Beach, USA; 2 Medicine, Edward Via College of Osteopathic Medicine, Spartanburg, USA

**Keywords:** abdominal blunt trauma, bilateral renal infarcts, hypoperfusion, renal injury, trauma-related vascular injury

## Abstract

Bilateral renal infarcts due to blunt trauma in the absence of underlying pathology are a rare condition. Trauma-induced renal infarction occurs due to decreased perfusion to the renal system. A 23-year-old woman with no significant past medical history presented to the emergency department with abdominal pain after being struck by a car as a pedestrian. She reported abdominal pain, shoulder pain, and pelvic pain. Imaging revealed multiple traumatic injuries, including a right shoulder dislocation, vertebral and pelvic fractures, and bilateral renal infarcts. Extensive workup, including echocardiography, renal ultrasound, magnetic resonance angiography, and hypercoagulability testing, was unremarkable. The findings were attributed to transient renal hypoperfusion secondary to trauma-related vascular dysfunction. Her symptoms resolved with conservative management, and she was discharged with outpatient follow-up. Renal infarcts are most commonly due to cardioembolic events, and trauma-induced renal infarcts are rare and typically result from hypoperfusion, vasospasm, or vascular injury. Contrast-enhanced CT imaging is used to diagnose renal infarcts. Most trauma-related cases of renal infarcts resolve spontaneously without any complications. This case highlights a rare instance of bilateral renal infarction after blunt abdominal trauma. Further research is warranted to discuss the clinical significance of and long-term outcomes of transient renal hypoperfusion in trauma patients.

## Introduction

Renal infarction is a rare but serious ischemic event that results from the occlusion of the main renal artery or its branches, potentially progressing to acute kidney injury, impaired glomerular filtration rate, end-stage renal disease, or death [1]. This condition can arise from various causes, the most common being cardiogenic [1]. Other causes, in no particular order, include renal artery injuries, hypercoagulable state, or idiopathic [1]. Despite its potentially severe clinical implications, renal infarction remains an uncommon diagnosis, with an estimated incidence of 0.004% among patients presenting to the Emergency Department [2]. The condition may often go unrecognized due to its nonspecific clinical presentation and overlap with more common causes of flank pain or abdominal discomfort. Although a rare condition, CT imaging with contrast, along with a detailed history and physical examination, is the best initial approach to identifying trauma-induced renal injury or a pre-existing renal pathology [3]. This case report presents an incidental discovery of bilateral renal infarcts in a trauma patient without any known predisposing medical conditions, including cardiac arrhythmia or coagulopathy. The absence of these risk factors underscores the importance of considering transient renal ischemic changes in the context of blunt abdominal trauma and highlights the need for further investigation into their pathophysiology and clinical implications.

## Case presentation

A 23-year-old woman with no significant past medical history presented to the emergency department as a Level 2 trauma activation after being struck by a car as a pedestrian. She experienced a brief loss of consciousness but was hemodynamically stable upon arrival, with mild tachycardia (Table 1) and a Glasgow Coma Scale (GCS) of 15. Primary survey revealed a patent airway, adequate breathing, and hemodynamic stability. Notable findings included a right shoulder dislocation and abrasions to the right shoulder. An Extended Focused Assessment with Sonography in Trauma (eFAST) exam was negative for free fluid. She endorsed abdominal pain, right shoulder pain, and left hip pain but denied dysuria, hematuria, fever, chills, nausea, vomiting, joint pain, or leg swelling.

**Table 1:** Pertinent vital signs of the patient upon admission to the Emergency Department BP: blood pressure; mmHg: millimeters of mercury; HR: heart rate; BPM: beats per minute; RR: respiratory rate; O2: oxygen

Variable	Value	Normal Range
Temperature	98.1 ˚F	97-99 ˚F
BP	115/79 mmHg	<120/80 mmHg
HR	116 bpm	60-100 bpm
RR	17	12-20 (breaths per minute)
Pulse Oximetry	98%	95-100% O2

The right shoulder dislocation was successfully reduced, and orthopedic evaluation was obtained with imaging revealing multiple traumatic injuries, including a nondisplaced left posterior iliac bone fracture, a left S1 transverse process fracture, and small avulsion fractures on the left calcaneus and navicular.

**Figure 1:**
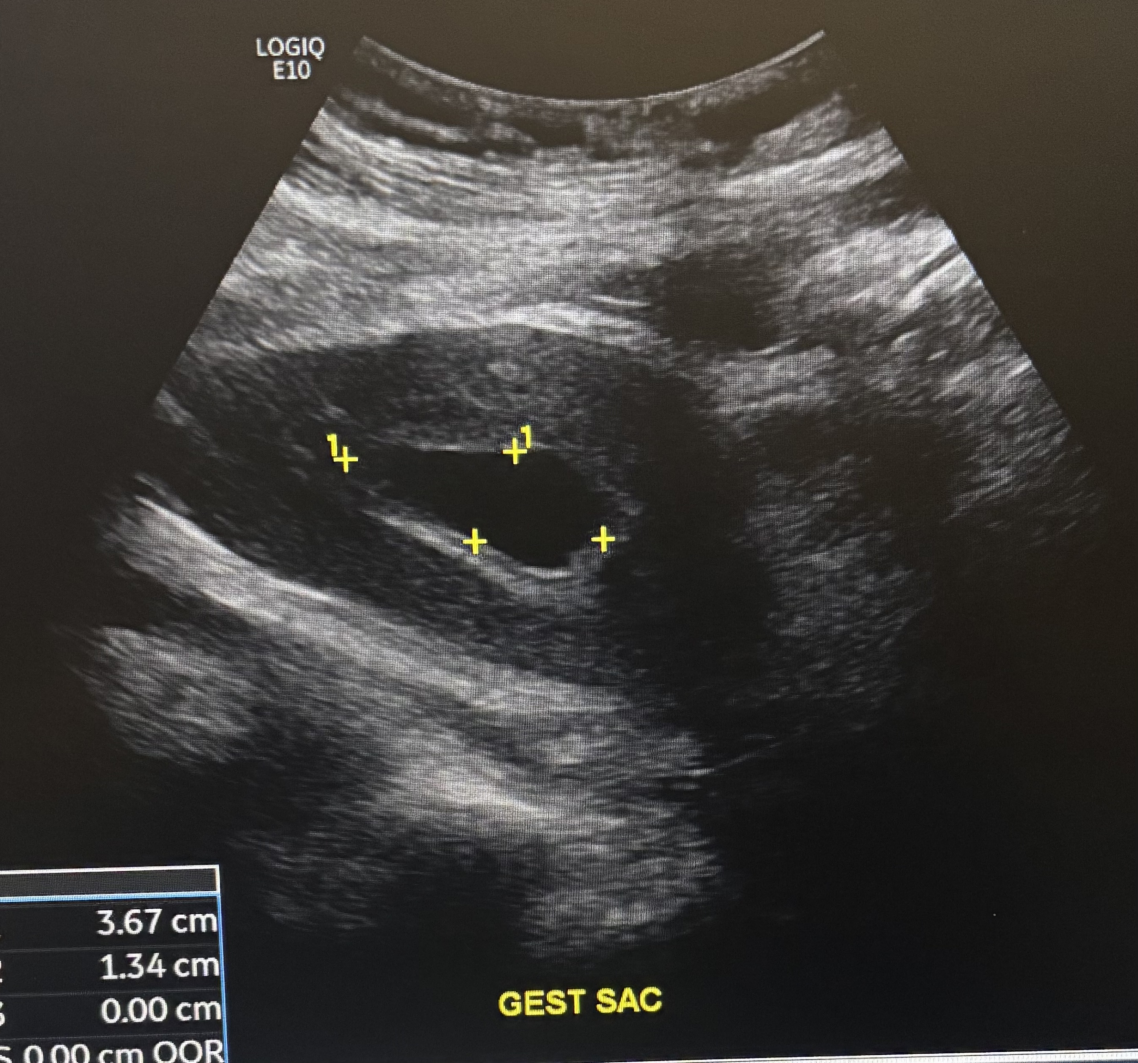
Ultrasound image of intrauterine pregnancy Ultrasound showing an intrauterine pregnancy with the gestational sac indicated by yellow markers

Obstetric and gynecologic (OBGYN) history included pre-eclampsia and a prior C-section at 37 weeks. She also had a history of gonorrhea and chlamydia. A transabdominal ultrasound identified an intrauterine pregnancy with a gestational sac (Figure 1) estimated at five weeks and six days with no gestational pole and possible small subchorionic hemorrhages (< 25%). She was diagnosed with a threatened abortion with a high likelihood of nonviability and scheduled a follow-up with her OBGYN upon discharge.

**Figure 2:**
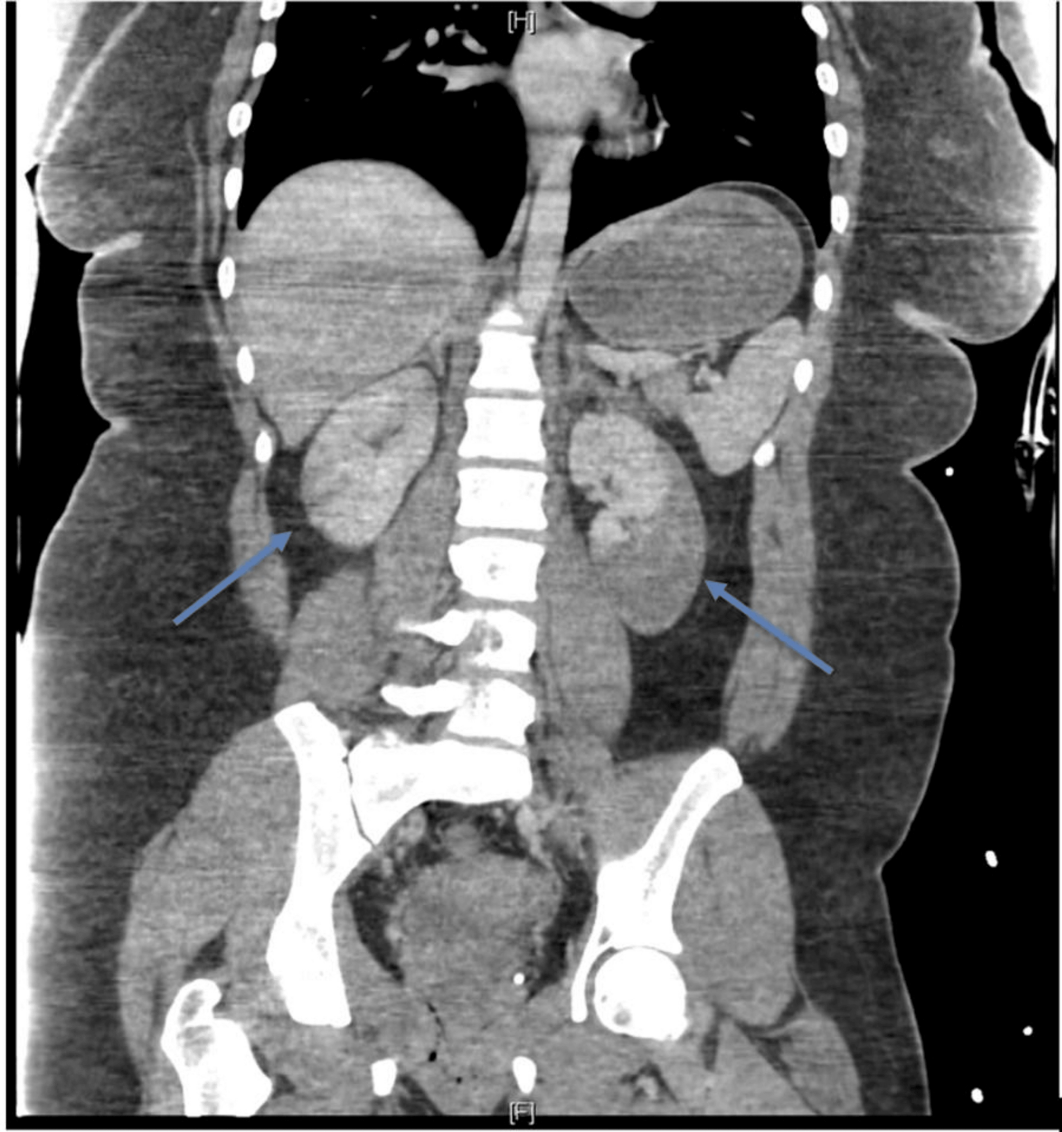
Contrast-enhanced coronal-view CT scan showing bilateral renal infarcts The blue arrows point to the infarcted right and left kidneys

**Figure 3:**
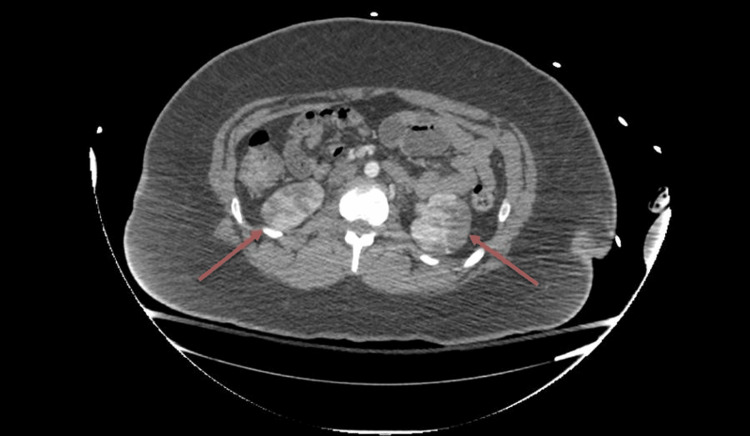
Contrast-enhanced axial-view CT scan also demonstrating bilateral renal infarcts The red arrows point to the right and left infarcted kidneys on the axial-view CT scan

Further imaging with contrast-enhanced computed tomography (CT) showed bilateral renal infarcts (Figures 2, 3) with a high suspicion for focal intimal injury in the main left renal artery, while the right renal artery appeared normal. These findings raised concern for a potential vascular injury from blunt trauma. Given the acute presentation and concern for serious underlying pathology, the patient was admitted for close monitoring and underwent an extensive diagnostic work-up to rule out vasculitis and cardioembolic etiologies. Initial laboratory tests, including complete blood count, basic metabolic panel, erythrocyte sedimentation rate, and urinalysis, were unremarkable. Additional testing, including echocardiogram, renal ultrasound, magnetic resonance angiogram of the abdomen, vasculitis panel, and hypercoagulable work-up, did not reveal any underlying pathology. There was no reported family history of renal disease or thrombophilia (Table 2). 

**Table 2:** Pertinent lab results excluding other causes of renal infarcts ANA: antinuclear antibody; c-ANCA: cytoplasmic antineutrophil cytoplasmic antibody; p-ANCA: perinuclear anti-neutrophil cytoplasmic antibodies; BUN: blood urea nitrogen; GFR: glomerular filtration rate; PT: prothrombin time; INR: international normalized ratio; APTT: activated partial thromboplastin time

Variables	Result	Units	Reference
Immunology Panel			
ANA	Negative		Negative
c-ANCA	<0.2	AI	0.0-0.9
p-ANCA	<0.2	AI	0.0-0.9
Complement C3	144	mg/dL	82-167
Complement C4	31	mg/dL	12-38
Kidney Function			
On Admission			
Creatinine	1.2	mg/dL	0.7-1.5
BUN	17	mg/dL	7-20
GFR	>=60		>=60
On Discharge			
Creatinine	0.9	mg/dL	0.7-1.5
BUN	15	mg/dL	7-20
GFR	>=60		>=60
Coagulation			
PT	12.5	seconds	9.8-13.9
INR	1.05		0.9-1.1
APTT	26.9	seconds	24.9-37.9

Given the absence of systemic causes, her renal hypoperfusion was considered secondary to transient hypoperfusion from blunt trauma. Throughout her hospitalization, she was managed conservatively with pain control, orthopedic monitoring, and urologic surveillance. Fluoroscopy exposure was limited due to pregnancy. She remained hemodynamically stable and was discharged with outpatient follow-up appointments scheduled with obstetrics and orthopedics.

## Discussion

Bilateral renal infarcts without an apparent etiology in a trauma patient represent a consequence of blunt abdominal trauma. While renal infarction may present with abdominal or flank pain, nausea, and vomiting, these symptoms are nonspecific and present in many other conditions [4]. Laboratory findings often include elevated lactate dehydrogenase levels, which serve as a marker of tissue ischemia, though our patient’s laboratory work-up was unremarkable.

Renal infarction can be caused by blockage of arterial or venous drainage; arterial blockage is more common than venous abnormalities [5]. Trauma-related renal infarction is a distinct entity and is a result of direct vascular injury, vasospasm, or transient hemodynamic instability that may contribute to renal hypoperfusion [6]. The most common cause of traumatic renal injury is decreased perfusion from hypotension caused by hemorrhage [7]. Blunt force trauma can cause endothelial damage, leading to vessel damage and infarction [8]. Additionally, vasospasm can occur after blunt trauma, similar to how it occurs in endovascular procedures. Vasospasm is a protective mechanism following vascular insult, which causes narrowing of the arteries, reducing renal perfusion [8,9].

Contrast-enhanced CT is the imaging modality of choice for diagnosing renal infarction, as early detection is important due to the short lifespan of the affected renal parenchyma [10]. The most common finding for renal infarct on CT imaging is a hypoattenuated area with an associated mass effect, followed by the “cortical rim sign”, which suggests preserved perfusion to the renal cortex via collateral vessels [5]. In this case, the bilateral nature of the infarcts and absence of a clear embolic or pathologic cause raises the suspicion of transient renal hypoperfusion due to trauma-related vascular dysfunction.

Trauma is the presumed etiology in this case; alternative causes of bilateral renal infarction must also be considered. Other etiologies include thromboembolic events, hypercoagulable states, or renal artery pathology [1,11]. The extensive work-up, including echocardiogram, vasculitis panel, and hypercoagulability testing, was unremarkable, making these alternative diagnoses less likely. Most trauma-induced renal infarcts resolve without long-term sequelae; some potential complications are chronic kidney disease, renal atrophy, or hypertension due to ischemic injury to the renal tissue [12,13]. Treatment of renal hypoperfusion can vary based on the presentation of the patient. Overall, the goal is to maintain adequate renal perfusion while avoiding fluid overload [14].

## Conclusions

Bilateral renal infarction identified on an abdominal contrast-enhanced CT scan without any underlying comorbid condition is an exceptionally rare finding, particularly in a healthy female patient following blunt abdominal trauma. This case highlights a benign and likely transient episode of bilateral renal hypoperfusion, detected incidentally through imaging performed during trauma evaluation. While symptoms were nonspecific, imaging modalities such as contrast-enhanced CT were critical in ruling out more concerning pathologies, including thromboembolic events and vascular injuries. The patient’s clinical course remained stable, with spontaneous resolution and no evidence of long-term renal impairment. This case underscores the importance of including renal hypoperfusion in the differential diagnosis following trauma and suggests that, in select cases after an appropriate work-up, conservative management with close monitoring is a safe and effective approach due to the potential for sequelae of more serious conditions.
